# Subcritical Water Chromatography with Electrochemical Detection

**DOI:** 10.3390/molecules22060962

**Published:** 2017-06-09

**Authors:** Heather Anderson, Yu Yang

**Affiliations:** Department of Chemistry, East Carolina University, Science & Technology Building 584, Greenville, NC 27858, USA; DUDLEYH@ecu.edu

**Keywords:** subcritical water chromatography, hot water chromatography, electrochemical detection, neurotransmitters, nucleic acids, heterocyclic bases, capsaicinoids

## Abstract

Reverse phase liquid chromatography (RPLC) is a commonly used separation and analysis technique. RPLC typically employs mixtures of organic solvents and water or aqueous buffers as the mobile phase. With RPLC being used on a global scale, enormous quantities of organic solvents are consumed every day. In addition to the purchasing cost of the hazardous solvents, the issue of waste disposal is another concern. At ambient temperature, water is too polar to dissolve many organic substances. Therefore, although water is nontoxic it cannot be used to replace the mobile phase in RPLC since organic analytes will not be eluted. Subcritical water chromatography may be an alternative. The characteristics of water, such as polarity, surface tension, and viscosity, can be altered by manipulating water’s temperature, thus making it behave like an organic solvent. The aim of this study was to evaluate the feasibility of separation using water mobile phase and detection by an electrochemical (EC) detector. The classes of analytes studied were neurotransmitters/metabolites, nucleic acids/heterocyclic bases, and capsaicinoids. Both isothermal and temperature-programmed separations were carried out. The separation temperature ranged from 25 to 100 °C. For separations of all three classes of solutes, the retention time was decreased with increasing temperature, thus shortening the analysis time. The peaks also became narrower as temperature increased. The limit of detection of neurotransmitters/metabolites ranges from 0.112 to 0.224 ppm.

## 1. Introduction

Organic solvents such as methanol and acetonitrile are widely employed in chromatographic separations. Being used on a global scale, enormous quantities of organic solvents are consumed every day. In addition to the cost of purchasing these toxic solvents, the waste has to be properly disposed of. With all these drawbacks in mind, a chromatographic solvent that is safe to the user and the environment is needed.

The above mentioned problems can be solved by using water as the mobile phase solvent. Water is widely available, nontoxic to the laboratory user, and environmentally benign. However, water under ambient conditions is too polar to dissolve many organic substances and thus cannot serve as a chromatographic eluent. Fortunately, this can be overcome. By manipulating the temperature of water, its properties can be tuned to mimic organic solvents [1,2,3]. This has led to an interest in the application of subcritical water in chromatography [2,3,4,5,6,7,8,9,10,11,12,13,14,15,16,17,18].

A commonly employed method of separation is reversed-phase liquid chromatography. This form of chromatography uses a nonpolar separation column with a polar mobile phase. The traditional RPLC mobile phase is typically a mixture of organic solvent(s) and water or aqueous buffers. When pure water at elevated temperatures and moderate pressures is used as the mobile phase to achieve RPLC separation, the technique is known as hot water chromatography or subcritical water chromatography. Under these conditions, water has properties that are typical to methanol/water and acetonitrile/water mixtures used in RPLC [1]. This gives hot water the potential to replace these organic solvent mixtures as the mobile phase in reverse phase separation [2,3,4,5,6,7,8,9,10,11,12,13,14,15,16,17,18]. 

A traditional RPLC system can easily be converted into a subcritical water chromatography system with the addition of a column heater and a back pressure regulator. Several different forms of detection methods have been employed in hot water chromatography with the most common form being ultraviolet (UV) detection [5,6,7,8,9,10]. Other detection techniques such as flame ionization detector [11,12,13,14] and NMR [15,16] have also been tested as the detector for subcritical water chromatography. However, UV can only detect analytes with chromophores. In this work, an electrochemical (EC) detector was added to a hot water chromatography-UV system. Either buffer only or a mixture of acetonitrile and buffer was used as the mobile phase. Three classes of solute were studied. The first two classes of analytes were biomolecules, neurotransmitters/metabolites and nucleic acids/heterocyclic bases. The third class was capsaicinoids, the substance found in red chili peppers that gives them hotness. A nonpolar C18 column was used as the stationary phase. This study was performed at temperatures ranging from 25 to 100 °C. Both isothermal and programmed temperatures were employed.

## 2. Results and Discussion

### 2.1. Separation of Neurotransmitters and Metabolites

#### 2.1.1. Isothermal Separations

**Figure 1:**
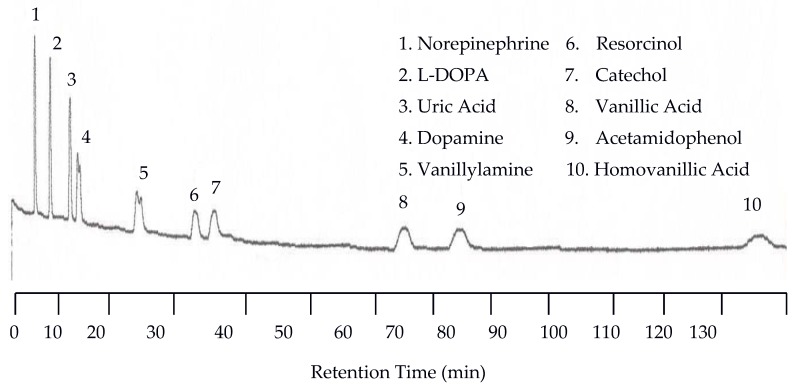
Water chromatogram of neurotransmitters and metabolites with electrochemical (EC) detection at 25 °C.

The separation of neurotransmitters was first performed at 25 °C with the UV detector set at 254 nm and the EC detector set at 0.750 V. An analyte mixture with 1.00 × 10^−5^ M was injected. Under these conditions no analytes were detected by the UV detector because of the lack of or weak chromophores of the analytes studied. On the other hand, the EC detector responded to each analyte, resulting in 10 peaks as shown in Figure 1. There are several problems with the chromatogram obtained at 25 °C. First, the separation took too long, over two hours. Second, the later eluting peaks are too broad. Third, dopamine and vanillyamine have double peaks.

**Figure 2:**
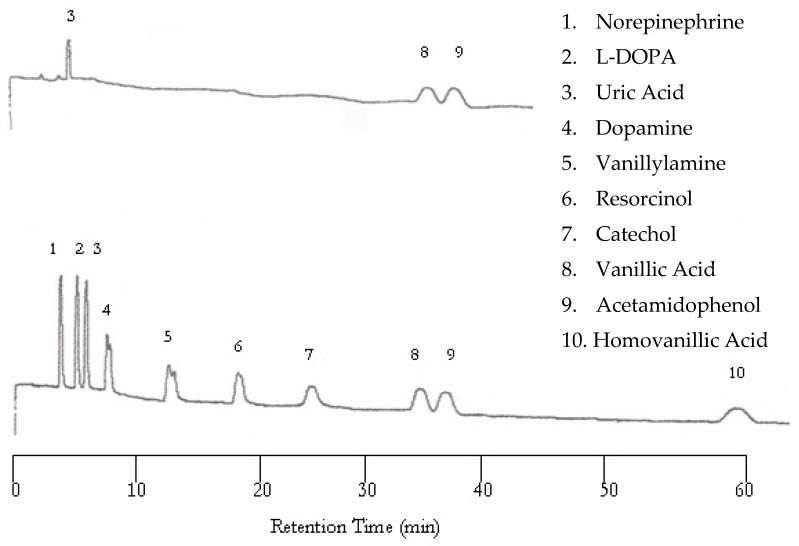
Water chromatograms of neurotransmitters and metabolites with both ultraviolet (UV) detection (**top**) and EC detection (**bottom**) at 50 °C.

The UV detector showed improvement upon increasing the temperature from 25 to 50 °C. The UV chromatogram now contains three peaks corresponding to uric acid, vanillic acid, and acetamidophenol, as shown in Figure 2 (top). It seemed that increasing temperature helped the compounds with weak chromophores to be detected. Again, the EC detector yielded a chromatogram with a peak for each analyte (Figure 2 bottom). Although the retention was shortened, dopamine and vanillyamine still have double peaks.

**Figure 3:**
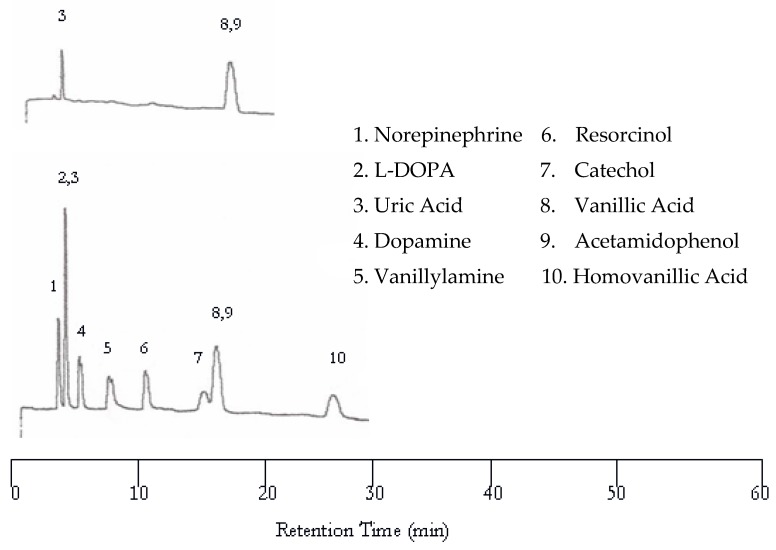
Water chromatograms of neurotransmitters and metabolites with both UV detection (**top**) and EC detection (**bottom**) at 75 °C.

Increasing the oven temperature to 75 °C resulted in co-eluted peaks as evidenced in Figure 3 while the retention time was significantly reduced. Like at 50 °C, only uric acid, vanillic acid, and acetamidophenol were detected by the UV detector. The other seven solutes were not detected by the UV detector due to the lack of chromophores. Only eight peaks are found in the EC chromatogram. The first co-eluted peak corresponds to L-DOPA and uric acid, while the second co-eluted peak corresponds to vanillic acid and acetamidophenol. The dopamine and vanillyamine peak shapes were improved at 75 °C, but the double peaks are still visible. 

**Figure 4:**
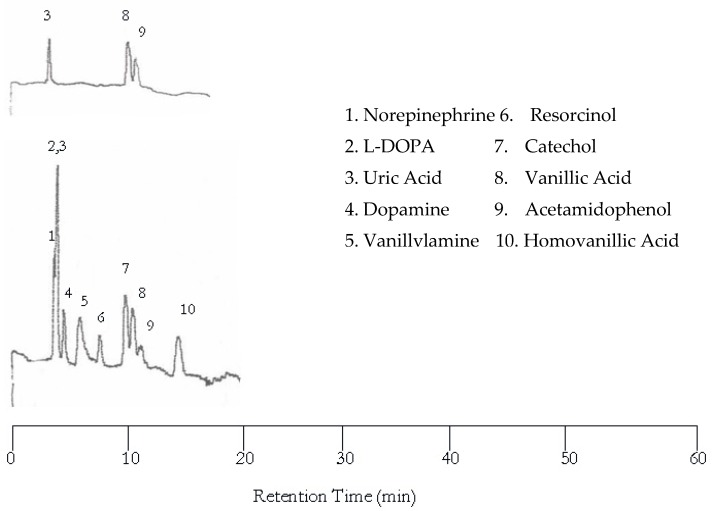
Water chromatograms of neurotransmitters and metabolites with both UV detection (**top**) and EC detection (**bottom**) at 100 °C.

Further increase in temperature to 100 °C resulted in more co-eluted peaks as shown in Figure 4 although the separation became much faster. Please note that the dopamine and vanillyamine double peak issue was resolved at 100 °C.

#### 2.1.2. Separation Using Temperature Programming

**Figure 5:**
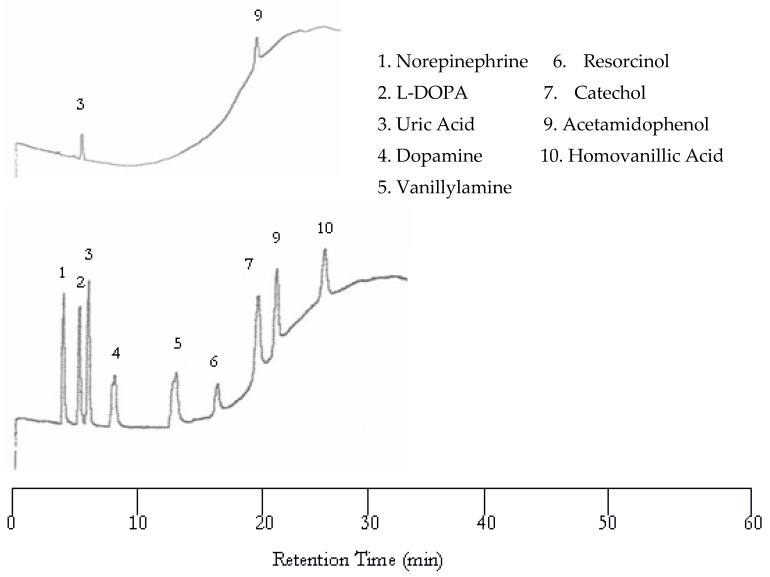
Water chromatograms of neurotransmitters and metabolites with both UV detection (**top**) and EC detection (**bottom**) using temperature programming.

To achieve better separation, a temperature program was created. The temperature at the beginning of the run was 50 °C. It was held at 50 °C for eight min, then increased to 100 °C at a rate of 5 °C per min. The temperature remained at 100 °C for the remainder of the run. Figure 5 shows the chromatogram using the above-mentioned temperature program. The only problem associated with this temperature programmed elution was the co-elution of vanillic acid with acetamidophenol. It was decided to remove vanillic acid from the analyte mixture. Although an increase in temperature caused the baseline to deviate from a flat line to a curve that is directly related to the increase of temperature, all nine peaks were well separated within 16 min in the EC chromatogram.

#### 2.1.3. Limit of Detection (LOD)

**Figure 6:**
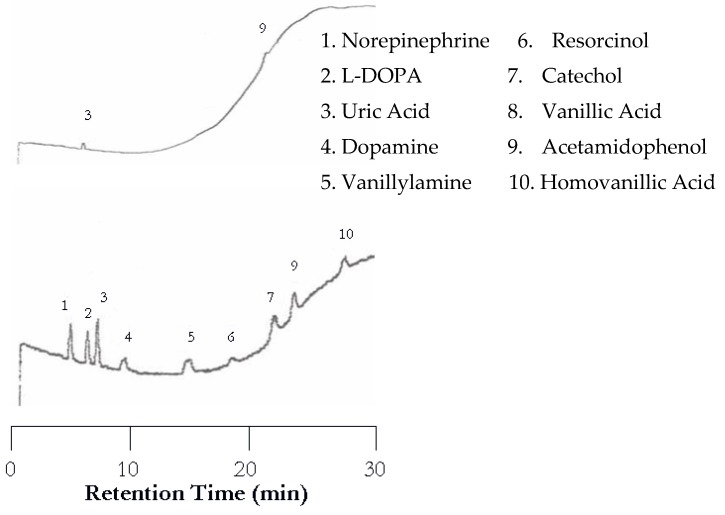
Water chromatograms of neurotransmitters and metabolites with UV detection (**top**) and EC detection (**bottom**) for a standard solution containing 0.224 ppm (resorcinol) to 0.408 ppm (norepinephrine) analytes.

**Figure 7:**
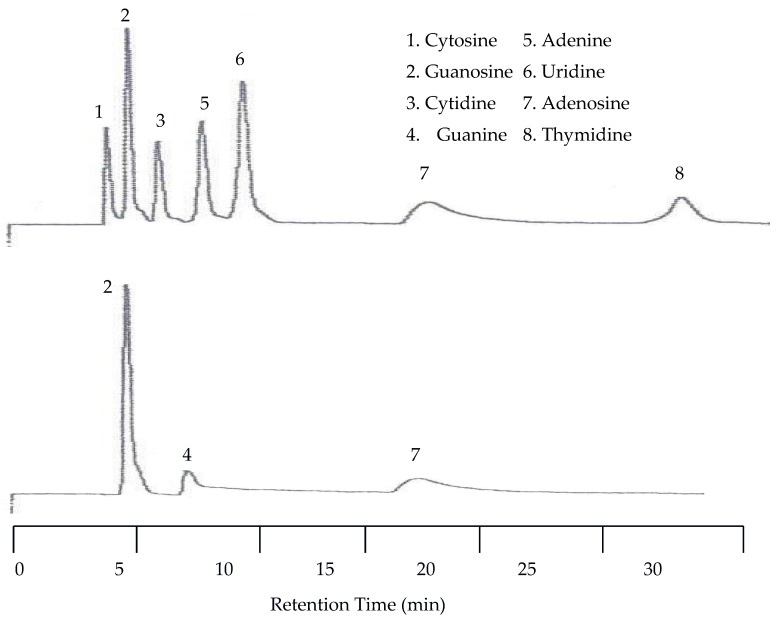
Water chromatograms of nucleic acids and heterocyclic bases with both UV detection (**top**) and EC detection (**bottom**) at 25 °C.

The limit of detection for neurotransmitters and metabolites was determined using elution with programmed temperature and diluted solutions. Figure 6 and Figure 7 show the chromatograms of two diluted test mixtures. LOD was estimated as the concentration obtained by a signal that equals or is greater than the blank plus three times standard deviation of the blank. The limit of quantification (LOQ) was estimated using the blank plus ten times standard deviation of the blank [19]. LOD and LOQ determined by the EC detector are given in Table 1.

### 2.2. Separation of Nucleic Acids and Heterocyclic Bases

#### 2.2.1. Isothermal Separations

The first separation of nucleic acids was performed at 25 °C. The UV detector was again set at 254 nm but the EC detector was set to 1.00 V. Unlike the neurotransmitters, the UV detector was superior to the EC for detecting nucleic acids and heterocyclic bases. Using an analyte mixture with approximately 5.00 × 10^−4^ M for each analyte, there were seven peaks detected by the UV detector, while only three were detected by the EC detector as seen in Figure 7. The benefit of EC detection is still applicable to this mixture as it is the only detector that showed a peak for guanine. 

**Figure 8:**
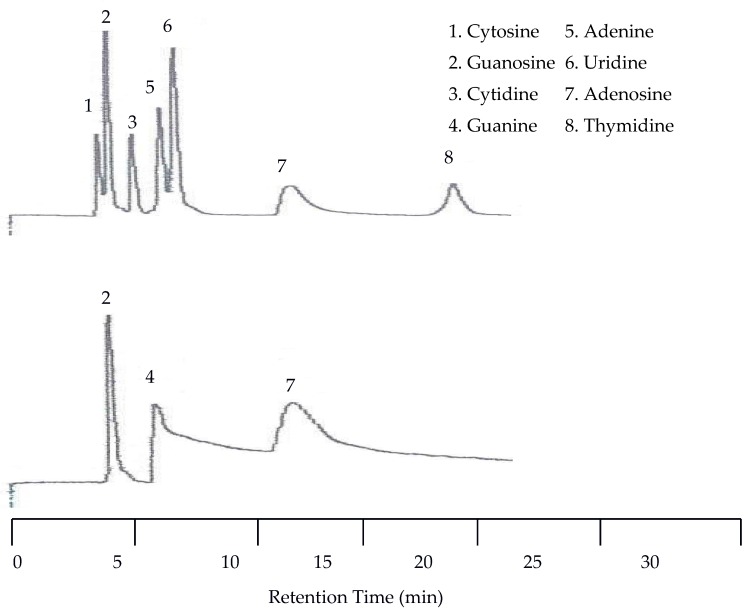
Water chromatograms of nucleic acids and heterocyclic bases with both UV detection (**top**) and EC detection (**bottom**) at 40 °C.

Since there was little spacing between peaks at 25 °C the next isothermal separation was only increased by 15 °C. With the temperature set to 40 °C all seven peaks were still detected by the UV detector. As seen in Figure 8, there was a slight co-elution between cytosine and guanosine as well as between adenine and uridine. There was no co-elution of the peaks with the EC detector but there was a slight upward drift of the baseline.

#### 2.2.2. Separation Using Temperature Programming

**Figure 9:**
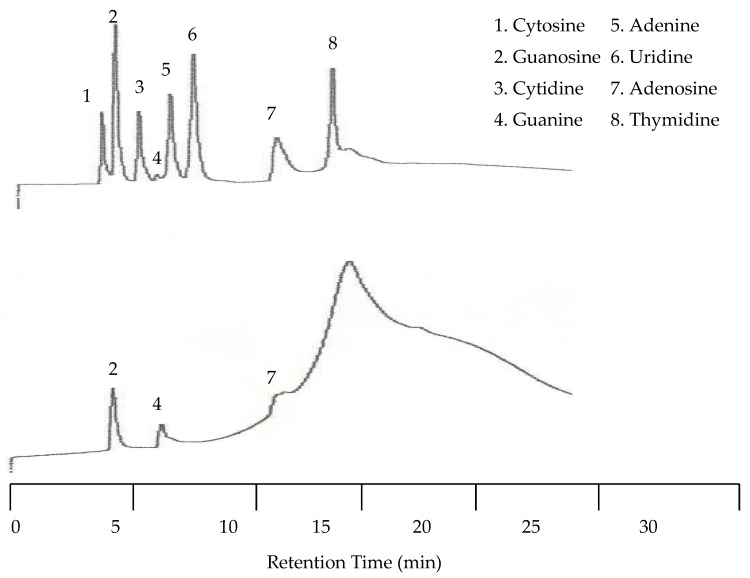
Water chromatograms of nucleic acids and heterocyclic bases with both UV detection (**top**) and EC detection (**bottom**) using temperature programmed elution.

Since co-elution was already observed at 40 °C, isothermal separations with increasing temperatures were discontinued and a temperature program was applied. This program started at 25 °C and was held at this temperature for eight min. Then the temperature was increased at a rate of 8 °C per min until 100 °C was reached. The remainder of the run was at 100 °C. Figure 9 shows that with temperature programming the appearance of a peak for guanine was observed by the UV detector for the first time. This resulted in a total of eight peaks detected by the UV detector. The EC detector again only detected three peaks. Unfortunately, at this increased voltage the temperature effects on the baseline for the EC detector were more dramatic.

### 2.3. Separation of Capsaicinoids

#### 2.3.1. Isocratic Separations

**Figure 10:**
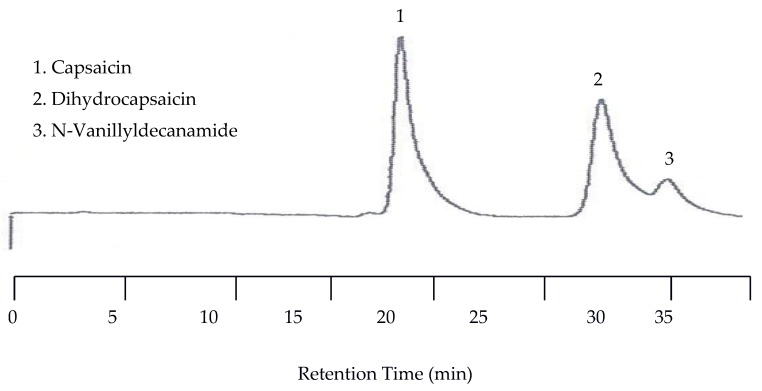
Chromatogram of capsaicinoids with EC detection at 25 °C with a 40:60 acetonitrile/buffer mobile phase.

In order to perform capsaicinoid separations, the mobile phase had to be mixed with an organic solvent. The solvent of choice was acetonitrile. In the first separation a mixture of capsaicin, dihydrocapsaicin, and N-Vanillyldecanamide was performed with a 55:45 acetonitrile/universal buffer mixture for the mobile phase. The temperature was set to 25 °C while the EC detector was set at 1.00 V. The wavelength of the UV detector was set to both 254 nm and 279 nm. Unfortunately, no peaks for any of the capsaicin analytes were detected by the UV detector due to the lack of chromophores. However, the EC chromatogram showed three peaks with the last two peaks co-eluted. The co-elution was improved when the amount of acetonitrile in the mobile phase was reduced to 40%. Figure 10 shows the chromatogram under this condition.

#### 2.3.2. Separations at Elevated Temperatures

**Figure 11:**
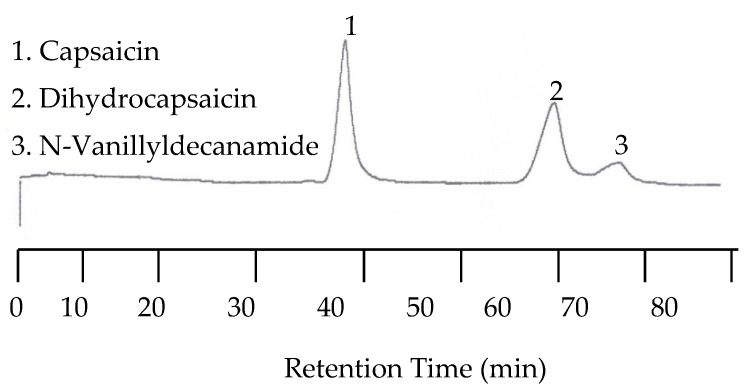
Chromatogram of capsaicinoids with EC detection at 80 °C and a 30:70 acetonitrile/buffer mobile phase mixture.

Because the last two peaks were not baseline resolved as shown in Figure 10, separation with a 30:70 acetonitrile/buffer mobile phase was carried out at 25, 50, 70, 80, 90, and 100 °C. While all three peaks were well separated at lower temperatures such as 25 and 50 °C, the separation took as long as 110 min at 50 °C. On the other hand, the retention time was significantly shortened, but co-elution occurred. Fortunately, the optimized separation was achieved at 80 °C, as shown in Figure 11.

## 3. Materials and Methods

### 3.1. Mobile Phase

The mobile phase employed in this study was either 100% universal buffer or a mixture of the universal buffer and acetonitrile (HPLC grade, Fisher Scientific, Pittsburgh, PA, USA). The buffer consisted of 0.025 M acetic acid, boric acid, and phosphoric acid (all from Fisher Scientific) each in 18 MΩ-cm water. The water was obtained from an in-house water purification system (SYBRON/Barnsted Co., Boston, MA, USA). The solution was then adjusted to a pH of 5 using 50% w/w sodium hydroxide (Fisher Scientific). The mobile phase was then filtered through a 0.2-μm nylon membrane (Alltech Associates, Inc., Deerfield, IL, USA). 

### 3.2. Reagents

The neurotransmitters and metabolites tested were acetamidophenol, catechol, L-DOPA, dopamine, vanillylamine, homovanillic acid, norepinephrine, resorcinol, vanillic acid (all from Aldrich Chemical, Milwaukee, WI, USA), and uric acid (Fisher Scientific). The working solution was prepared in universal buffer with a concentration of 1.12–2.04 ppm (approximately 1 × 10^−5^ M) for each of the 10 compounds listed above. 

The second class of analytes was nucleic acids and heterocyclic bases including adenine, adenosine, cytidine, cytosine, guanine, guanosine, thymidine, and uridine (all from Aldrich Chemical, Milwaukee, WI, USA). The working solution was prepared in universal buffer with a concentration of 56–204 ppm (approximately 5.00 × 10^−4^ M) for each of the eight analytes in this group. 

The capsaicinoids used were capsaicin, dihyrocapsaicin, and N-vanillylnonanamide (VANA) (all from Sigma Chemical, St. Louis, MO, USA). The working solution was prepared in acetonitrile with a concentration of 148–152 ppm (approximately 5.00 × 10^−4^ M) for each of the three analytes listed above.

### 3.3. Subcritical Water Chromatography System

**Figure 12:**
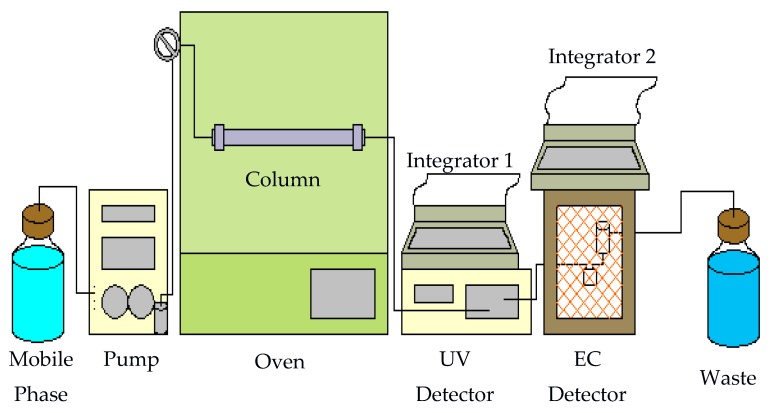
Subcritical water chromatography system.

A homemade system used to perform hot water chromatographic separation is shown in Figure 12. The mobile phase was delivered by an LDC pump (constaMetric, LDC Analytical, Riviera Beach, FL, USA). The outlet of the pump was connected to a Valco injector with a 20-μL sample loop (Keystone Scientific, Inc., Bellefonte, PA, USA) using stainless steel tubing (58 cm × 0.005 in i.d.). The injector was located outside an Isotemp 800 series programmable oven (Fisher Scientific). Stainless steel tubing (35 cm) was used again to connect the injector to the column which was housed inside the oven. The column used was a Chromatorex-C18 column (250 × 4.6 mm i.d., 5 μm, Fuji Silysia, Raleigh, NC, USA). This was followed by 55 cm of stainless steel tubing leading to a SPD-10AVP UV detector (Shimadzu, Columbia, MD, USA). Another 20 cm of stainless steel tubing connected the UV detector followed by an electrochemical (EC) detector. The EC detector is a BAS LC-4B amperometric detector.

The EC detector consists of a thin-layer electrochemical flow cell. The flow cell is comprised of two half-blocks. The bottom is a KEL-F half-block and the top half-block is stainless steel. The glassy carbon electrode is embedded in the KEL-F block. Electrical connection is made through a gold pin extending from the bottom of the block. A 0.005-in Teflon spacer is placed between the two blocks forming an oval-shaped thin-layer flow space. The flow volume through the space is approximately 10 μL. The two blocks and spacer are held together using four screws. The stainless steel block has an inlet port that connects it to the UV detector. Flow travels across the working electrode and then through the inlet port of the reference electrode compartment. A silver/silver chloride reference electrode, which is held in place by a rubber O-ring, is manually inserted into the top of this compartment. The outlet port of this compartment is stainless steel, allowing use as a counter electrode. The outlet also leads to the waste container. All electrodes are housed in a Faraday cage to block environmental noise. HP 3396 Series II integrators were used for data acquisition (Hewlett-Packard, Avondale, PA, USA).

### 3.4. Subcritical Water Chromatographic Separation

Prior to each use the EC detector flow cell was disconnected and disassembled. The stainless steel half-block and Teflon spacer are rinsed with 18 MΩ-cm water. The other half-block with the glassy carbon electrode was polished with a coarse powder in a figure eight motion on a camel hair polishing pad. This was followed by polishing with a fine powder and sonication in18 MΩ-cm water for 30 s. Polishing should occur not only daily but also every time the applied potential is changed. After sonication the electrode was reassembled and reattached to the system.

The mobile phase was degassed with pure helium for 15 min. The mobile phase was then purged through the pump at 5 mL/min to remove any residual air. After all air bubbles were removed, the flow rate was lowered to 1 mL/min and the mobile phase was directed through the system. When the mobile phase reached the reference electrode compartment the Ag/AgCl electrode was inserted. The gold pin was also inserted into the working electrode. The UV detector was turned on and set to the appropriate wavelength. The three connection wires for the EC detector were attached and the appropriate voltage was applied. An initial surge of current was observed and a steady decay occurred until the system reached equilibrium. This generally took about 1 h. If heating or temperature programming was used in a run, the oven was turned on 30 min into the decaying process. 

When the output on the EC detector has reached a steady state, the system is ready to perform a run. First the output was adjusted to zero. Then the injector was set to the load position and overloaded with the analyte sample to ensure that exactly 20 μL was in the sample loop. The injector was switched to the inject position while turning on both integrators at the same time. The system continues to run until all desired peaks are seen, whereupon both integrators are stopped. If another run is desired, the injector was returned to the load position and the above steps are repeated from output adjustment onward. After the completion of all runs, the oven was turned off and 18 MΩ-cm water was run through the system at 1 mL/min until the system returned to room temperature. Then the pump, UV detector and EC detector were all turned off. The gold pin for the working electrode was removed for safe keeping, while the Ag/AgCl electrode was removed and stored in 4.00 M KCl solution. 

## 4. Conclusions

Because UV cannot detect analytes without chromophores, an EC detector was coupled to a homemade subcritical water chromatography system in this study. As we expected, the use of EC detection enabled the detection of analytes without chromophores. The first class of analytes in this study, 10 neurotransmitters and their metabolites were separated using only buffer as the mobile phase isothermally at temperatures ranging from 25 to 100 °C. While all 10 analytes were separated at 25 and 50 °C, the elution of homovanillic acid required 130 and 60 min at 25 and 50 °C, respectively. There are double peaks for dopamine and vanillylamine at both temperatures. The separation became much faster at 75 and 100 °C, however, co-elution was observed at these two temperatures. The best separation of neurotransmitters and their metabolites was achieved using programed temperature, although the base line was not ideal. The detection limit ranges from 0.112 for catechol to 0.224 ppm for resorcinol. 

Although all eight nucleic acids and heterocyclic bases were separated at 25 °C, the separation was slow and the later eluting peaks were too broad. While the elution time was shortened and peaks became narrower at 40 °C, the earlier peaks were co-eluted. The best separation of nucleic acids and heterocyclic bases was obtained by temperature programmed elution although the EC chromatogram’s baseline went up. 

Capsaicinoids are the only class of analytes studied that required the presence of an organic solvent in the mobile to achieve chromatographic separation. The optimized separation conditions for capsaicinoids are 30:70 acetonitrile:buffer as the mobile phase and at 80 °C. Please note that one can also optimize the column to achieve more efficient separation for capsaicinoids. 

With regards to detection, neither UV nor EC detection was so beneficial that it ruled out the other for the analytes studied. Both can be used in subcritical water chromatography but both have drawbacks with particular analytes. The characteristics of the analyte should be considered when choosing a detection method. 

## Figures and Tables

**Table 1:** EC detection limit of neurotransmitters and metabolites. LOD: limit of detection; LOQ: limit of quantification.

Peak Number	Analyte	LOD (ppm)	LOQ (ppm)
1	Norepinephrine	0.204	0.680
2	L-DOPA	0.196	0.647
3	Uric Acid	0.168	0.554
4	Dopamine	0.152	0.502
5	Vanillylamine	0.188	0.620
6	Resorcinol	0.224	0.739
7	Catechol	0.112	0.370
9	Acetamidophenol	0.152	0.502
10	Homovanillic Acid	0.184	0.607
